# Chronic Lymphocytic Leukemia: Prognostic Factors at Presentation in a Resource-Limited Center

**DOI:** 10.1200/GO.20.00276

**Published:** 2021-01-12

**Authors:** Kaladada Ibitrokoemi Korubo, Uchechukwu Prince Okite, Sampson Ibekwe Ezeugwu

**Affiliations:** ^1^ Department of Haematology & Blood Transfusion, University of Port Harcourt Teaching Hospital, Port Harcourt, Nigeria

## Abstract

**PURPOSE:**

Determining chronic lymphocytic leukemia (CLL) prognosis using the International Prognostic Index markers such as TP53 and immunoglobulin heavy-chain variable region gene mutation in a resource-limited setting is difficult to achieve because of cost and equipment unavailability. The aim of this study is to determine prognostic factors easily available to hematologists in low- or medium-income countries.

**MATERIALS AND METHODS:**

This was a retrospective study conducted at the University of Port Harcourt Teaching Hospital, Nigeria. Data were retrieved from CLL patient records from January 2004 to December 2019 (15 years). Data collected were analyzed using SPSS software version 25.

**RESULTS:**

A total of 46 records were reviewed, with a median age of 55 years and a male:female ratio of 1:1.2. All patients were symptomatic at presentation, with splenomegaly (91.3%), anemia (82.6%), and lymphadenopathy (76.1%) predominating. About 89.1% of the patients presented at Binet stage C and/or high-risk Rai (Rai stages III and IV) with 10.9% presenting at Binet stage B and/or intermediate-risk Rai (Rai stage II). Only 13% of the patients had immunophenotyping done with 6.5% being done for the Matutes CLL score. The 5-year overall survival (OS) was 15.7% with a median survival of 26 months. WBC count and absolute lymphocyte count (ALC) > 100 × 10^9^/L were significant poor prognostic markers (*P* = .013 and .021, respectively). Thirty-five (76.1%) received chemotherapy, and they had a better median survival than those who did not (26 *v* 17.5 months). The most common regimen used was cyclophosphamide, vincristine, and prednisolone for 15 (42.9%) patients.

**CONCLUSION:**

WBC count and ALC > 100 × 10^9^/L were poor prognostic markers. Patients who received chemotherapy had a better OS.

## INTRODUCTION

Chronic lymphocytic leukemia (CLL) is a clonal B cell malignant disease with attendant lymphocytosis characterized by progressive accumulation of clonal B cells in the blood, bone marrow, lymph nodes, and spleen permitting easy diagnosis from the peripheral blood. The clinical features of CLL at presentation are related to the accumulation of these leukemic cells.

**CONTEXT**

**Key Objective**
Prognostic factors such as del(11q), del(17p)/TP53, and immunoglobulin heavy-chain variable region gene mutation are unavailable in many resource-limited settings. What other available factors could aid prognostication in patients diagnosed with Chronic Lymphocytic Leukemia?
**Knowledge Generated**
Patients with WBC count and absolute lymphocyte count (ALC) > 100 × 10^9^/L at presentation had a poor prognostic outlook when compared with those with WBC count and ALC < 100 × 10^9^/L (*P* = .013 and .021, respectively).
**Relevance**
A majority of patients could not afford novel drugs such as ibrutinib, obinutuzumab, or venetoclax; hence, older management protocols such as CP (chlorambucil and prednisolone), CHOP (cyclophosphamide, hydroxydaunorubicin, oncovin, and prednisolone), and CVP (cyclophosphamide, vincristine, and prednisolone) were used. Patients on CHOP appeared to have a better prognostic outlook.

CLL is predominantly a disease of the elderly, with a median age of 65-72 years at diagnosis.^1,2^ It is a slowly progressive disease, with an 81.7% 5-year survival rate.^1^ It accounts for 17%-20% of all hematologic malignancies in Nigeria with 2-6 per 100,000 new cases diagnosed annually.^3,4^ CLL accounts for one quarter of the new cases of leukemia with an age-adjusted incidence of 4.5 per 100,000 persons in the United States.^1^ The incidence varies widely across geographical locations, with Asia having a 5-10-fold lower prevalence.^5,6^ It is more common in males, mostly seen in the elderly (age > 65 years) with some familial tendency being alleged.^2,7^

Diagnosis requires peripheral blood lymphocytosis, an absolute lymphocyte count (ALC) of ≥ 5 × 10^9^, and clonality confirmed by immunophenotyping (IMPT). Staging of the tumor is usually done at diagnosis, using Rai or Binet staging,^2^ both of which are used for prognosis and decision on the commencement of therapy. The system uses lymphadenopathy, organomegaly, and cytopenias (anemia and thrombocytopenia) to establish prognostic groups that can be used to predict median survival. The Rai and Binet staging systems were the first prognostic markers to affect disease management. Although still widely used, they do not predict disease progression or response to therapy. There are numerous ongoing efforts to identify and characterize additional prognostic markers at the cellular and molecular level.^1,2,8^

Serum β_2_-microglobulin (β_2_m), thymidine kinase, and soluble CD23 have all been described as independent prognostic markers in CLL.^9,10^ Other newer prognostic factors include mutational status of the immunoglobulin heavy chain variable region (IGVH) genes and surrogate markers such as CD38 and zeta-associated protein (ZAP)-70 gene expression, telomerase length, and telomerase activity.^9,11^ In a resource-limited setting like Nigeria, most of these investigations cannot be done and hence the need to investigate the usefulness of some clinical and laboratory parameters in assessing the prognosis and survival of patients with CLL.

Not all patients require treatment at diagnosis. Indications for treatment include a rapid doubling time, worsening cytopenias and symptomatic nodal or extranodal disease. Treatment options include the use of alkylating agents, purine analogs, anti-CD20 monoclonal antibodies, Bruton Tyr kinase inhibitors, and BCL-2 inhibitors.^12^

The aim of this study was to identify some clinical and laboratory prognostic markers for patients with CLL in a resource-limited setting.

## MATERIALS AND METHODS

This was a retrospective study conducted at the University of Port Harcourt Teaching Hospital. Data were retrieved from the files of all patients diagnosed with CLL from January 2004 to December 2019. Inclusion criteria were asymptomatic and symptomatic patients with ALC > 5 × 10^9^/L and peripheral blood film showing predominantly mature lymphocytes with a Matutes score of 4 or 5 (if IMPT was done). Those with ≥ 10% prolymphocytes on blood film or Matutes score ≤ 3 were excluded. Data collected were analyzed using SPSS software version 25 (IBM, Armonk, NY). The results were expressed in charts and tables. A *P* value of < 0.05 (at 95% CI) was taken as statistically significant.

## RESULTS

A total of 46 patients (making up 21.1% of 218 hematological malignancies) were diagnosed with CLL from January 2004 to December 2019 (15 years). There were 21 (45.7%) males and 25 (54.3%) females with a male to female ratio of 1:1.2. The median age at presentation for all the cases was 55 years (range, 39-83 years). Twenty-four patients (52.2%) were ≤ 55 years at presentation. Females had a lower median age at presentation (55 years) compared to the males (58 years); however, this was not statistically significant (*P* = .35).

**FIG 1:**
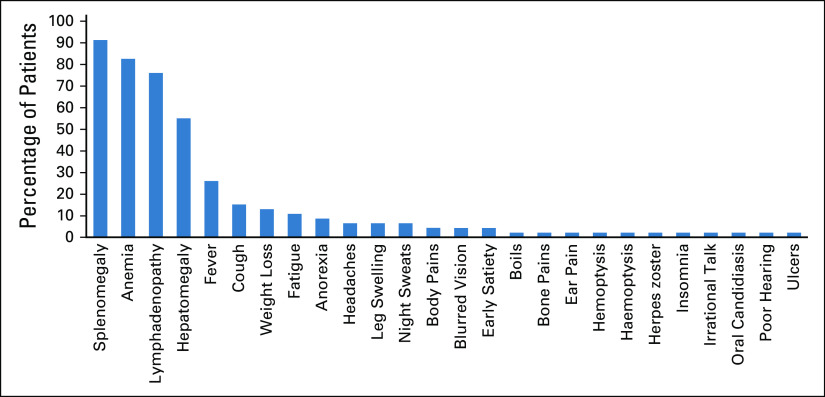
Clinical features at presentation.

There was no asymptomatic case, and the median duration of symptoms was 5 months (range, 3 weeks to 168 months). The most common symptoms were splenomegaly seen in 42 (91.3%) of the cases, followed by anemia (n = 38, 82.6%) and lymphadenopathy (n = 35, 76.1%). A little over a quarter of them (n = 12, 26.1%) had fever. The mean spleen size was 14 cm (± 6.7 cm). Of the 42 patients with splenomegaly, 6 (14.3%) had a spleen size > 20 cm (massive splenomegaly). One patient presented with asplenia following splenectomy for hypersplenism. Figure 1 shows a summary of the clinical features of the patients with CLL.

The median packed cell volume (PCV), total WBC count, ALC, and platelet count were 24% (range, 8.7%-39%), 101.0 × 10^9^/L (range, 26.3-642.0 × 10^9^/L), 88.7 × 10^9^/L (range, 21.3-622.7 × 10^9^/L), and 131.5 × 10^9^/L (12-392 × 10^9^/L), respectively. Using the PCV and platelet count cutoff from the Rai staging criteria, there were 33 patients (71.7%) with anemia and 18 (39.1%) with thrombocytopenia. Eighteen patients (39.1%) presented at Rai stage 4, whereas 23 (50%) presented at Rai stage 3 and 5 (10.9%) at Rai stage 2. None of the patients presented at Rai stage 0 or 1 or Binet stage A, 41 patients (89.1%) presented at Binet stage C, and 5 (10.9%) at Binet stage B.

IMPT was done for only six patients (13.0%); half of them (n = 3, 50%) had only CD20 done, whereas the other three (50%) were tested using the Matutes CLL score. All the six cases who had IMPT done were CD20+ (Table 1).

**TABLE 1:**
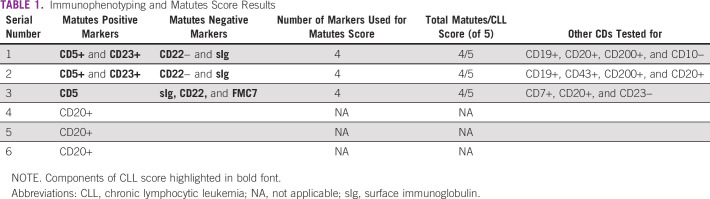
Immunophenotyping and Matutes Score Results

All 46 patients had indications for treatment: 35 (76.1%) received chemotherapy and 11 (23.9%) did not receive chemotherapy (of these, 2 [18.1%] patients were referred to chemotherapy and 4 [36.4%] died before commencing treatment, whereas 5 [45.5%] refused to undergo chemotherapy). The mean duration of treatment for all regimens was 4.2 months (± 3.2 months). The major first-line chemotherapy used was cyclophosphamide, vincristine, and prednisolone (CVP) for 15 patients (42.9%), followed by chlorambucil and prednisolone (CP) for 11 (31.4%). Only three (8.6%) patients had rituximab with cyclophosphamide, hydroxodaunorubicin, oncovin, and prednisolone (R-CHOP). Fludarabine, cyclophosphamide, and prednisolone (FCP) was received by two patients (Table 1).

Patients on chemotherapy were grouped into those who received older forms of therapy (such as CP, CVP, and cyclophosphamide, hydroxodaunorubicin, oncovin, and prednisolone [CHOP]; n = 30, 85.7%) and those who received newer therapy, which were either FCP or R-CHOP (n = 5, 14.3%). Patients who received older therapy had a median survival of 26 months, whereas those who had newer therapy had a median survival of 38 months, although this was not statistically significant (*P* = .8). Patients who had CHOP had the longest median survival of 48 months (Table 2). The median survival was 26 months for all patients, with a 3-year overall survival (OS) of 47.2%, whereas the 5-year OS was 15.7%. Females had a better median survival than males (26 months *v* 13 months); however, this was not statistically significant (*P* = .3). Survival rate of younger patients (≤ 55 years) was better than patients of age > 55 years (29.9% *v* 11.08%), *P* = .9. Patients with massive splenomegaly (> 20 cm) had a shorter median survival (6 months) compared with those with splenomegaly < 20 cm (26 months), although this was not statistically significant, *P* = .3.

**TABLE 2:**
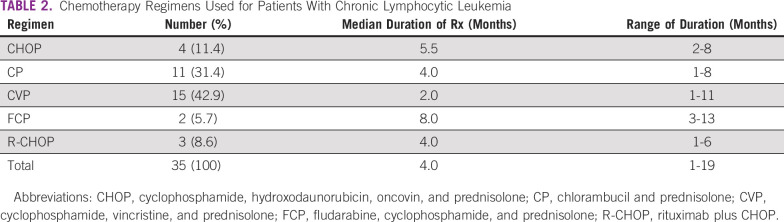
Chemotherapy Regimens Used for Patients With Chronic Lymphocytic Leukemia

**FIG 2:**
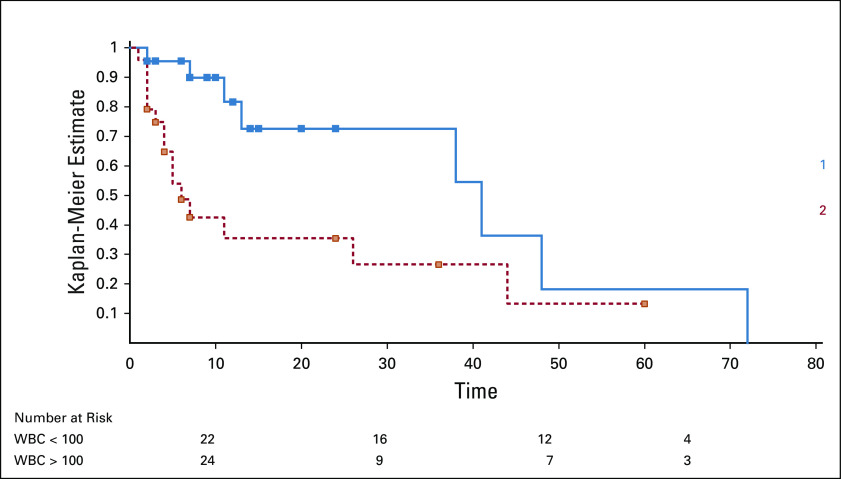
Survival curve showing the survival rates of those with WBC count < 100 × 10^9^/L (1, blue line) compared with WBC count > 100 × 10^9^/L at presentation (2, red line) (Note: Time in months).

Patients with WBC count > 100 × 10^9^/L (n = 24, 52.2%) had a significantly lower 5-year survival rate of 13.3% (a median survival of 41 months) compared with those with WBC count < 100 × 10^9^/L (n = 22, 47.8%) whose 5-year OS was 18.2% (a median survival of 6 months), which was significant (*P* = .013) (Fig 2, Table 3). Also, patients with ALC > 100 × 10^9^/L (n = 22, 47.8%) had a significantly lower median survival of 6 months compared with those with ALC < 100 (41 months), which was also significant (*P* = .021).

**TABLE 3:**
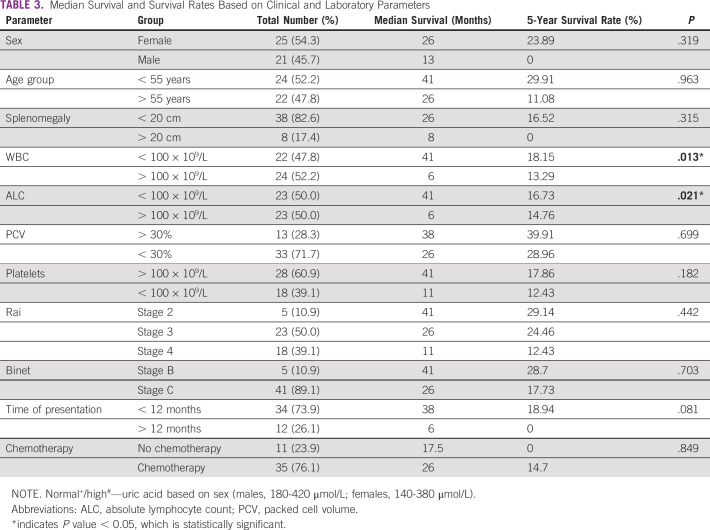
Median Survival and Survival Rates Based on Clinical and Laboratory Parameters

Patients who received chemotherapy had a higher median survival of 26 months with a 5-year survival rate of 14.7%, those without chemotherapy had a median survival of 17.5 months, and 5-year survival rate was zero. The median follow-up duration for all patients was 5 months (range, 0.5-72 months).

## DISCUSSION

The median age at diagnosis of CLL was 55 years, showing a younger age at presentation when compared with other studies.^1,2^ Salawu et al^13^ showed a median age of 60 years in Ile-Ife, South-West Nigeria. However, western studies have shown a median age of between 65 and 72 years.^1,2,14^ More than half of our patients with CLL were young (< 55 years) compared with those in the United States and Europe where only about 5%-11% of cases are younger than 55 years.^14,15^ Although the epidemiology of CLL both in Nigeria and globally generally shows a male predominance,^16^ our study showed a slight female preponderance. A female preponderance has also been reported in our environment.^17^

No patient was asymptomatic, unlike in western studies where 25%-50% of patients are asymptomatic at presentation.^18^ This could be due to the poor health seeking behavior and the low socioeconomic status of the populace. This is further buttressed by the long median duration of symptoms. More than 90% of our patients had splenomegaly, which is higher than the results found by Basabaeen et al in Sudan and Salawu et al in Nigeria (49.1% and 70.9%, respectively).^12,19^ Patients with massive splenomegaly had a lower median survival, albeit not statistically significant.

Slightly more than half of the patients presented with WBC count and ALC > 100 × 10^9^/L, representing a high tumor burden, possibly associated with bone marrow suppression (because anemia was seen in more than three quarters of them, whereas thrombocytopoenia was present in more than a third of the cases). The presence of anemia and thrombocytopoenia explains why a majority had a high-risk Rai (III and IV) and Binet C at the time of presentation.

Only 13% of our patients could afford the cost of IMPT, of which half of them tested for the Matutes CLL score (CD5, CD23, FMC7, surface immunoglobulin, and CD22/CD79b) and the remaining half were tested for CD20 only. IMPT should be performed routinely as part of the workup for diagnosis of CLL as this aids confirmation of diagnosis and excludes other lymphoid neoplasms whose blood or marrow morphology may mimic CLL.^2^

The median survival was 26 months, which is only slightly above 2 years. This is poor when compared with some western studies like the one conducted by Weide et al,^20^ in which the median survival from the time of diagnosis was 12.3  years for patients diagnosed in 2001 or earlier and 13.3  years for patients diagnosed between 2002 and 2008, whereas the median survival was not achieved in those diagnosed between 2009 and 2017. This short median survival may be due to misdiagnosis of patients who may have had other more severe lymphoproliferative neoplasms, since IMPT was not routinely performed. Our study showed that young patients < 55 years had a better OS than those > 55 years, even though it was not statistically significant. This is similar to studies where patients younger than 55 years had better OS than older patients.^14,15^ The better survival in the younger age group (despite having more poor prognostic markers such as ZAP-70 expression or unmutated immunoglobulin heavy-chain variable status) could be due to their fewer comorbidities and shorter time to treatment, when compared with the older age group.^15^ The females in our study had a slightly younger median age than males (55 years *v* 58 years), which may have contributed to the better median survival than the males (26 months *v* 13 months).

Patients with WBC count and ALC < 100 × 10^9^/L had a statistically significant better median survival than those with higher WBC counts or ALC, suggesting that a high tumor burden at presentation is a poor prognostic feature. This is validated by other studies that showed similar results.^9,21^ However, Madu et al showed that there was no association between the ALC and median survival.^14^ The hematocrit, platelet count, and Rai and Binet staging did not have any statistically significant impact on OS in our patients, unlike several other studies where these factors were associated with a shorter OS, with the exception of Salawu et al who noted that their patients with anemia had a better median OS.^2,22^

The later the stage at presentation using Rai or Binet, the shorter the median survival. Since our patients presented late, this was not statistically significant in our findings but might have been due to not having patients who presented with Rai 0 or I or Binet-A, which is yet to be compared. Other studies have shown a similar pattern in median survival, with it declining at later stages of presentation (Rai stage 0 > 150 months; stage I, 101 months; stage II, 71 months; stages III and IV, 19 months each).^13^ Patients with Binet-A had a longer median OS of 100 months; stage B patients, 55 months; and stage C patients, 45 months.^23^ Our patients with Rai stages II, III, and IV had the median survival of 41, 26, and 11 months, respectively. None of our patients presented in Binet-A, but patients with Binet-B had a better median survival than those at stage C.

Patients who received chemotherapy had a higher median survival than those who did not (26 *v* 17.5 months). A majority of our patients were not exposed to newer regimens because of nonavailability and financial implications. Although CVP was the most used older regimen, patients who received CHOP had the best median survival. This is similar to the 1989 findings by the French Cooperative Group on CLL.^24^ In recent years, CLL treatment has evolved. First-line regimens now include novel drugs such as BTK inhibitors (ibrutinib), newer anti-CD20 monoclonal antibodies (obinituzumab), or BCL-2 inhibitors (venetoclax).^12^ Therefore, comparatively speaking, our five patients who had the newer regimens did not *actually* receive novel therapy, and this may explain the poor survival in our patients with CLL compared with other climes. Those five patients in the newer therapy group (FCP and R-CHOP) had better median survival unlike the older therapy group (38 *v* 26 months); however, patients on CHOP had the longest median survival (Table 4).

**TABLE 4:**
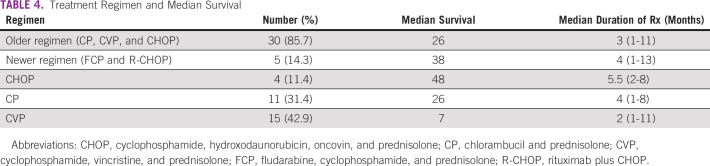
Treatment Regimen and Median Survival

In conclusion, while striving for better diagnostic and therapeutic availability and reduced costs, there is a need to use the limited available investigations and drugs at our disposal to patients with prognosticate CLL in our environs. Our patients presented at a younger median age of 55 years. At presentation, WBC count and ALC > 100 × 10^9^/L are poor prognostic markers. Patients who received chemotherapy had a longer median survival. It is recommended that full blood count is done for all patients for earlier detection of CLL, which may affect survival.

## References

[1] Rai KR Jain P: Chronic lymphocytic leukemia (CLL)-Then and now. Am J Hematol 91:330-340, 2016 26690614 10.1002/ajh.24282

[2] Hallek M: Chronic lymphocytic leukemia: 2017 update on diagnosis, risk stratification, and treatment. Am J Hematol 92:946-965, 2017 28782884 10.1002/ajh.24826

[3] Nwannadi IA Alao OO Bazuaye GN et al: The epidemiology of haematological malignancies at the University Of Benin teaching hospital: A ten-year retrospective study. Int J Epidemiol 9, 2010

[4] Egesie OJ Agaba PA Silas OA et al: Presentation and survival in patients with hematologic malignancies in Jos, Nigeria: A retrospective cohort analysis. J Med Trop 20:49-56, 2018 29963503 10.4103/jomt.jomt_8_18 PMC6024253

[5] Yang SM Li JY Gale RP et al: The mystery of chronic lymphocytic leukemia (CLL): Why is it absent in Asians and what does this tell us about etiology, pathogenesis and biology? Blood Rev 29:205-213, 2015 25541495 10.1016/j.blre.2014.12.001

[6] Kipps TJ Stevenson FK Wu CJ et al: Chronic lymphocytic leukaemia. Nat Rev Dis Primers 3:16096, 2017 28102226 10.1038/nrdp.2016.96 PMC5336551

[7] Goldin LR Slager SL Caporaso NE: Familial chronic lymphocytic leukemia. Curr Opin Hematol 17:350-355, 2010 20389242 10.1097/MOH.0b013e328338cd99 PMC2891437

[8] Fischer K Hallek M: Optimizing frontline therapy of CLL based on clinical and biological factors. Hematology Am Soc Hematol Educ Program 2017:338-345, 2017 29222276 10.1182/asheducation-2017.1.338 PMC6142543

[9] Gowda A Byrd JC: Use of prognostic factors in risk stratification at diagnosis and time of treatment of patients with chronic lymphocytic leukaemia. Cur Opin Haematol 13:266-272, 2006 10.1097/01.moh.0000231425.46148.b0 16755224

[10] Gentile M Cutrona G Neri A et al: Predictive value of beta2-microglobulin (beta2-m) levels in chronic lymphocytic leukemia since Binet A stages. Haematologica 94:887-888, 2009 19483161 10.3324/haematol.2009.005561 PMC2688585

[11] Eichhorst B Hallek M: Prognostication of chronic lymphocytic leukemia in the era of new agents. Hematology Am Soc Hematol Educ Program 2016:149-155, 2016 27913474 10.1182/asheducation-2016.1.149 PMC6142472

[12] Sharma S Rai KR: Chronic lymphocytic leukemia (CLL) treatment: So many choices, such great options. Cancer 125:1432-1440, 2019 30807655 10.1002/cncr.31931

[13] Salawu L Bolarinwa R Durosinmi M: Chronic lymphocytic leukaemia: A-twenty-years experience and problems in Ile-Ife, South-Western Nigeria. Afr Health Sci 10:187-192, 2010 21326974 PMC2956293

[14] Parikh S Rabe K Kay N et al: Chronic lymphocytic leukemia in young (≤ 55 years) patients: A comprehensive analysis of prognostic factors and outcomes. Haematologica 99:140-147, 2014 23911703 10.3324/haematol.2013.086066 PMC4007929

[15] Delgado J Villamor N: Chronic lymphocytic leukemia in young individuals revisited. Haematologica 99:4-5, 2014 24425687 10.3324/haematol.2013.096297 PMC4007945

[16] Madu AJ Korubo K Okoye A et al: Presenting features and treatment outcomes of chronic lymphocytic leukaemia in a resource poor Southern Nigeria. Malawi Med J 31:144-149, 2019 31452848 10.4314/mmj.v31i2.7 PMC6698622

[17] Omoti C Imiere E: Trends in the pattern of leukaemia incidence in a tertiary health center in Nigeria: 1990-2004. J Med Biomed Res 5:44-49, 2006

[18] Parker T Strout M: Chronic lymphocytic leukemia: Prognostic factors and impact on treatment. Discov Med 11:115-123, 2011 21356166

[19] Basabaeen AA Abdelgader EA Babekir EA et al: Clinical presentation and hematological profile among young and old chronic lymphocytic leukemia patients in Sudan. BMC Res Notes 12:202, 2019 30940190 10.1186/s13104-019-4239-7 PMC6446286

[20] Weide R Feiten S Chakupurakal G et al: Survival improvement of patients with chronic lymphocytic leukemia (CLL) in routine care 1995–2017. Leuk Lymphoma 61:557-566, 2020 31682164 10.1080/10428194.2019.1680840

[21] Montserrat E Rozman C: Chronic lymphocytic leukaemia: Prognostic factors and natural history. Baillieres Clin Haematol 6:849-866, 1993 8038493 10.1016/s0950-3536(05)80179-9

[22] Dhodapkar M Tefferi A Su J et al: Prognostic features and survival in young adults with early/intermediate chronic lymphocytic leukemia (B-CLL): A single institution study. Leukemia 7:1232-1235, 1993 8350623

[23] Apelgren P Hasselblom S Werlenius O et al: Evaluation of clinical staging in chronic lymphocytic leukemia- population-based study. Leuk Lymphoma 47:2505-2516, 2006 17169795 10.1080/10428190600881322

[24] Long-term results of the CHOP regimen in stage C chronic lymphocytic leukaemia. French Cooperative Group on Chronic Lymphocytic Leukaemia. Br J Haematol 73:334-340, 19892690923

